# Moderating effects of coping strategies on the associations between stigma and depression among emerging adults

**DOI:** 10.1016/j.jad.2025.119843

**Published:** 2025-07-10

**Authors:** Tao Lin, Mingcong Tang, José A. Bauermeister, Jennifer T. Tran, Marin M. Kautz, Hayoung Donnelly, Jessica Webster, James R. Wolfe, Jennifer Ben Nathan, Amanda Arcamano, Maria A. Oquendo, Gregory K. Brown, David Mandell, Danielle Mowery, Lily A. Brown

**Affiliations:** a Department of Psychiatry, University of Pennsylvania, United States of America; b Department of Family and Community Health, School of Nursing, University of Pennsylvania, United States of America; c Department of Biostatistics, Epidemiology and Informatics, University of Pennsylvania, United States of America

**Keywords:** Coping, Discrimination, Stigma, Depression, Emerging adults

## Abstract

**Backgrounds:**

Emerging adult sexual and gender minority (EA-SGM) populations experience many forms of stigma, which places them at greater risk for mental health problems. In this prospective study, we examined the association between depression and three types of stigma, gender identity stigma, discrimination, and enacted stigma in EA-SGM. We also examined whether different coping strategies moderated these associations.

**Methods:**

We used longitudinal data collected from 64 EA-SGM as part of a pilot randomized controlled trial. Participants’ mental health was assessed at baseline, 2 months, 4 months, and 6 months. We used time-lagged, mixed-effects models to examine the moderating effects of three coping strategies (i.e., problem-focused, avoidant, and emotion-focused coping) on the association between stigma and subsequent depression.

**Results:**

Problem-focused coping buffered the negative effects of gender identity stigma on depression but did not protect against the effects of discrimination and enacted stigma. In contrast, avoidant coping exacerbated the association between discrimination and depression. None of the three coping strategies moderated the association between enacted stigma and depression.

**Conclusion:**

The moderating effects of coping strategies varied by type of stigma. If these findings are confirmed, future mental health interventions for minority populations may benefit from tailored coping strategies to specific stressors.

## 1. Introduction

Emerging adult sexual and gender minority (EA-SGM) populations are at heightened risk for mental health problems, including depression, anxiety, and suicidal ideation, compared to their cisgender heterosexual peers (Brown et al., 2023; Dolsen et al., 2022). EA-SGM individuals experience depression and anxiety at rates nearly double those of the general population, with up to 40% reporting depressive symptoms and 30% experiencing serious psychological distress (Poteat et al., 2021). Emerging adulthood is a key developmental stage marked by increased vulnerability to mental health challenges. This period can be especially challenging for SGM individuals because they experience pervasive stigma related to their sexual or gender identity and often lack the necessary resources and support to navigate these adversities (Alessi et al., 2023).

Minority Stress Theory (Frost and Meyer, 2023; Meyer, 2003) posits that EA-SGM encounter many forms of stigma related to their marginalized status, especially for those who belong to other marginalized groups, such as racial or ethnic minorities (Veenstra, 2011). This stigma, in turn, increases susceptibility to adverse mental health outcomes (Vargas et al., 2020), particularly depression (Alessi et al., 2023; Källström et al., 2022). Stigma differs in its internal versus external focus, degree of controllability, and level of severity and has distinct effects on mental health (Puckett and Levitt, 2015; Vargas et al., 2020). Three specific forms of stigma— *gender identity stigma*, *discrimination*, and *enacted stigma*—have unique relevance to EA-SGM and may confer increased risk for mental health symptoms, though more research is needed on this topic.

Gender identity stigma refers to internalized negative attitudes about gender identity (Freeland et al., 2018). Gender identity stigma can lead to self-stigmatization, compromised self-esteem, self-criticism, and internalized shame (Falck and Bränström, 2023; Hughto et al., 2015; Maksut et al., 2020; Mizock and Mueser, 2014). Adjusting negative self-perception and accepting identity may be essential to address gender identity stigma given its internalized nature (Oorthuys et al., 2022). However, little is known about the direct effect of gender identity stigma on depression, nor about the extent to which different coping styles alter this association.

Discrimination refers to external and systemic experiences of being unfairly treated due to one or more marginalized identities, such as being denied employment or healthcare (Schmitt et al., 2014). Unlike interpersonal stigma that occurs in social interactions (e.g., being mocked by a peer), discrimination often occurs within institutional or systemic settings and is structural in nature. Discriminatory experiences can contribute to feelings of isolation and diminished self-worth (Pascoe and Richman, 2009; Schmitt and Branscombe, 2002). LGBTQ-based discrimination has a direct effect on depression symptoms among LGBTQ people of color (Sutter and Perrin, 2016). To counter these experiences, individuals may benefit from coping strategies that directly address the unfair treatment or, in situations when little can be done to change the discrimination, to reappraise these experiences to reduce the toxic impact on the target of the discrimination (Duker et al., 2022; Gabarrell-Pascuet et al., 2023; Salcido and Stein, 2024). For example, for a person who was denied employment, instead of personalizing this experience; it may be helpful to engage in reappraisal and interpret the unfairness as a failing of the source of the discrimination as opposed to a personal failing.

Enacted stigma is another form of external stigma, but involves interpersonal experiences of overt and aggressive actions, such as physical assault or sexual harassment (Freeland et al., 2018). Enacted stigma can have immediate and tangible impacts on mental health by reinforcing the reality of external threats (Falck and Bränström, 2023; Veale et al., 2017) and has been consistently linked to depression (Lo Hog Tian et al., 2021). Coping with enacted stigma may require active engagement focusing on protecting oneself from harm and resilience-building, as well as seeking out safe spaces and social support (Varni et al., 2012; Veale et al., 2017). While enacted stigma is an established predictor of depression, little research has been conducted on this topic among EA-SGM and no research has explored whether different coping styles moderate the effect of enacted stigma on depression.

Minority stress theory identifies multiple types of stressors that are broadly categorized into distal and proximal stressors (Frost and Meyer, 2023; Meyer, 2003). Distal stressors are external and objective experiences, such as discrimination and enacted stigma, while proximal stressors are internal, subjective processes, such as gender identity stigma, including internalized shame and negative self-evaluation (Timmins et al., 2020). These stressors follow different pathways in impacting mental health, and the same response may result in divergent and even opposite effects depending on the stressor type. For example, coming out may reduce proximal stress but increase exposure to distal stressors such as discrimination (Timmins et al., 2020). Gender identity stigma, discrimination, and enacted stigma may require distinct coping strategies given their unique characteristics and varying impacts on individuals. Studies indicate that EA-SGM individuals who experience multiple forms of stigma exhibit more severe depressive symptoms (Mereish et al., 2021; Vargas et al., 2020). Identifying coping strategies tailored to each stigma type is crucial for developing targeted, personalized interventions to alleviate the psychological burden on this vulnerable population.

Coping (or its absence) can buffer or exacerbate the mental health impacts of stigma (Berjot and Gillet, 2011; Forster et al., 2022; Gabarrell-Pascuet et al., 2023). Three common coping strategies include problem-focused, emotion-focused, and avoidant coping (Baker and Berenbaum, 2007; Ben-Zur, 2009). Problem-focused coping involves actively addressing the source of stress, such as seeking solutions, gathering information, or directly confronting a stressor (DeLongis and Newth, 2001). Problem-focused coping may help EA-SGM individuals feel proactive, reducing internalized negativity and potentially buffering the adverse effects of stgima on mental health (Mizock and Mueser, 2014). Avoidant coping includes denial, distraction, or substance use, and tends to involve efforts to disengage from stressors entirely (Balmores-Paulino, 2018). While avoidance may offer temporary relief from emotional distress, it often exacerbates mental health issues in the long term, especially in the context of pervasive stigma (Holahan et al., 2005; Schibalski et al., 2017). For EA-SGM individuals, avoidant coping might increase psychological distress by leaving the stressor unad-dressed and reinforcing feelings of isolation. Emotion-focused coping involves managing emotional responses to stress rather than directly addressing its source (Stanisławski, 2019; Zhaoyang et al., 2019), and includes emotional support, acceptance, seeking social support, practicing self-care, or engaging in mindfulness (Ben-Zur, 2017; Carver, 1997). The extent to which these coping styles moderate the effect of stigma on mental health among EA-SGM is unknown.

Coping strategies may influence mental health outcomes differently depending on the type of stigma individuals face (Berjot and Gillet, 2011). For instance, by empowering individuals to seek solutions or support and change their circumstances, problem-focused coping can reduce feelings of helplessness and bolster resilience (Isaksson et al., 2017). Emotion-focused coping, often through social support or self-acceptance, has been found to be beneficial when changing external conditions is not feasible (Oorthuys et al., 2022). According to the goodness-of-fit hypothesis (Conway and Terry, 1992; Park et al., 2012; Roubinov et al., 2015), problem-focused coping is often effective in situations where an individual has control over the outcome, while emotion-focused coping may be more suitable when the stressor is beyond the individual’s control (Palamarchuk and Vaillancourt, 2021). For instance, applying problem-focused strategies to uncontrollable external stressors—such as systemic discrimination—may result in frustration or a sense of failure if those efforts are unsuccessful. Over time, this mismatch may amplify distress and reduce the perceived effectiveness of future coping efforts. As such, problem-focused coping may be especially effective for gender identity stigma given its internalized, controllable nature.

Avoidant coping has been linked to worsened mental health especially when coping with enacted stigma, as avoidance tends to exacerbate feelings of fear and isolation rather than address the underlying stressor (Rehr and Nguyen, 2022; Zhang et al., 2022). These findings suggest that coping strategies are not universally beneficial and may have varying effects depending on the type of stigma experienced. Understanding how diverse coping strategies interact with different types of stigma can provide valuable insight into protective factors that could mitigate depression among EA-SGM individuals.

However, previous findings on the interaction between coping and stigma are mixed, making it challenging to draw consistent conclusions. Previous coping research has primarily focused on a single type of stigma instead of investigating multiple types of stigma simultaneously. Given the variations in study designs across studies, including differences in the minority populations studied (e.g., racial minorities, sexual minorities) as well as inconsistent definitions and measures of stigma (Vargas et al., 2020), it is challenging to synthesize these mixed findings and fully understand how coping strategies interact with distinct stigma experiences to influence mental health outcomes. Most previous coping research relies on cross-sectional data, limiting understanding of how coping strategies interact with distinct types of stigma to influence mental health outcomes over time.

To address these research gaps, this study examined the associations between three types of stigma—gender identity stigma, discrimination, and enacted stigma—and depression in EA-SGM. Using longitudinal data, this project explored how different coping strategies (problem-focused, emotion-focused, and avoidant) moderate these relationships over time. First, we hypothesized that problem-focused coping would buffer the negative impacts of stigma on depression. Second, we hypothesized that avoidant coping would exacerbate the negative effects of stigma on depression. Third, we had an exploratory hypothesis that the moderating roles of coping strategies would vary across different types of stigma.

## 2. Methods

This study uses data from a pilot randomized controlled trial (clinicaltrials.gov registration: NCT05018143), Supporting Transitions to Adulthood and Reducing Suicide (STARS), the goal of which was to reduce suicidal ideation and behaviors among EA-SGM in a northeast metropolitan area of the United States (Brown et al., 2023).

### 2.1. Participants and recruitment

Participants were recruited through ads on social media platforms (e. g., Facebook, Instagram, Grindr) and in community spaces in the Greater Philadelphia area. Potential participants completed a screener survey through REDCap where initial eligibility was assessed based on the following criteria (Harris et al., 2009): a) emerging adults aged 18–24 years, b) self-identification as a sexual and/or gender minority, c) lived in the Philadelphia metro area, d) owned and had regular access to a smartphone, e) reported having suicidal ideation in the prior month, and f) denied self-reported psychosis symptoms. The final sample consisted of 64 EA-SGM (see Table 1 for the summary of the demographic characteristics). All 64 EA-SGM were included in the analysis.

### 2.2. Procedures

Study procedures were approved by the IRB of the University of Pennsylvania (protocol # 849500). Following consent and enrollment, participants attended an in-person visit with a research team member and the study clinician where they completed a survey, were administered the Columbia-Suicide Severity Rating Scale, Screening Version (C-SSRS; Posner et al., 2011), and received the Safety Planning Intervention (SPI; Stanley et al., 2018; Stanley and Brown, 2012). Participants were then randomized in a 1:1 ratio to either the control condition (SPI only) or the intervention condition (SPI plus access to the STARS app and six peer mentor sessions). Participants were assessed for their mental health outcomes at baseline, and 2, 4, and 6 months and were compensated $50 for the baseline, $30 for the 2-month assessment, $40 for the 4-month assessment, and $50 for the 6-month assessment. More detailed information on the clinical trial can be found in the published protocol paper (Brown et al., 2023).

### 2.3. Measures

#### 2.3.1. Minority score

Each participant was assigned a value (1–4) to reflect the possession of one or more minority identities: racial, ethnic, sexual, and gender. Participants who self-reported belonging to any race other than White received a point in the racial minority category. Participants who reported being Hispanic, Latino, Latinx, or of Spanish heritage received a point in the ethnic minority category. Participants who reported any sexual identity other than straight received a point for the sexual minority category. Participants who reported a gender identity other than cisgender received a point for gender minority.

#### 2.3.2. Coping

We used the Brief-COPE, a 28-item self-report questionnaire, to measure effective and ineffective ways to cope with a stressful life event (Carver, 1997). Participants answered questions about different ways of coping (for example: “I’ve been getting emotional support from others”) on a scale from “1” = “I haven’t been doing this.” to “4” = “I’ve been doing this a lot.” In our study, we focus on three subscales of interest: problem-focused coping, emotion-focused coping, and avoidant coping. All subscales demonstrated acceptable internal reliability (Cronbach’s α = 0.63–0.84).

#### 2.3.3. Gender identity stigma

Gender identity stigma was measured using an adapted version of the Identity subscale from the Collective Self-Esteem Scale (Luhtanen and Crocker, 1992). Example adapted items include: “My gender identity is an important reflection of who I am” and “I worry that people will judge my appearance negatively.” Participants answered all five items on a 4-item Likert scale (1 = strongly disagree to 4 = strongly agree). Scores ranged from 1 to 4, with higher scores indicating higher levels of gender identity stigma. Previous research has shown that this adapted subscale has good reliability and convergent validity (McLemore, 2018). In our sample, the internal reliability was acceptable (Cronbach’s α = 0.74).

#### 2.3.4. Discrimination

Discrimination experiences were assessed using an adapted scale of major experiences of discrimination scale (Williams et al., 2008). The scale contains 8 items asking whether participants have experienced different forms of discrimination. An example question is, “Have you ever been denied employment or fired from a job?” Participants answer these questions on a scale from 1 = Never to 4 = Often. Higher scores indicate higher levels of discrimination. In our sample, the internal reliability was good (Cronbach’s α = 0.80). Furthermore, the discrimination scale score was significantly correlated with the Everyday Discrimination Scale (*r* = 0.464, *p* = .002), a well-established scale of perceived discrimination, suggesting high convergent validity (Williams et al., 1997).

#### 2.3.5. Enacted stigma

Enacted stigma was assessed utilizing an adapted checklist on experiences of enacted stigma with sexual minority populations (Herek, 2007). Participants are presented with five items, with an example question asking, “Please indicate how often they have experienced each incident during their lifetime: been threatened with physical violence.” Participants answer on a 4-point scale of 1 = never to 4 = often. Higher scores indicated higher levels of enacted stigma. In our sample, the internal reliability was acceptable (Cronbach’s α = 0.72). The enacted stigma scale score was significantly correlated with the Everyday Discrimination Scale score (*r* = 0.539, *p* < .001), suggesting high convergent validity.

#### 2.3.6. Depression

Depression was assessed using the Center for Epidemiologic Studies Depression Scale Revised (CESD-R; Eaton et al., 2004). This scale is a 10-item self-report measure. A sample item is, “I was bothered by things that usually don’t bother me.” Participants answered items on a scale of Rarely or none of the time (<1 day) to All of the time (5–7 days). Scores of 10 or more are considered a cutoff score for depression. In our sample, the internal reliability was good (Cronbach’s α = 0.83).

### 2.4. Data analysis

Data analyses were performed using R 4.3.3 version (R Core Team, 2024). First, we estimated bivariate correlations to examine the associations among coping, stigma, and depression (Supplemental Table 1). Next, to examine the effects of three types of discrimination and stigma on future depression and whether coping strategies moderated this association, we conducted a time-lagged mixed-effects model using the lme4 package in R for each of the three types of stigma (Bates et al., 2015). To facilitate interpretation of the results, coping and stigma variables were standardized. We started with an unconditional model. The dependent variable was depression at time *T*_n+1._ The independent variables were stigma at time *T*_n_, problem-focused coping, emotion-focused coping, and avoidant coping. Depression at time *T*_n_, minority status, and group condition were included as covariates. The interaction terms between gender identity stigma and the three coping strategies were then added. Below is the formula for the full model:

$${\text{Depression}}_{T_{n+1}}=\beta_{0}+\beta_{1}\text{Group}\;\text{Condition}\;+\beta_{2}\text{Minority}\;\text{Score}\;+\beta_{3}{\text{Depression}}_{T_{n}}+\beta_{4}{\text{Stigma}}_{T_{n}}+\beta_{5}\text{Problem}\;\text{focused}\;\text{Coping}+\beta_{6}\text{Emotion}\;\text{focused}\;\text{Coping}\;+\beta_{7}\text{Avoidant}\;\text{Coping}+\beta_{8}{\text{Stigma}}_{T_{n}}\times \text{Problem}\;\text{focused}\;\text{Coping}\;+\beta_{9}{\text{Stigma}}_{T_{n}}\times \text{Emotion}\;\text{focused}\;\text{Coping}\;+\beta_{10}{\text{Stigma}}_{T_{n}}\times \text{Avoidant}\;\text{Coping}\;+u_{0}+e$$

To illustrate the interaction effects, we performed simple slope analyses for the relationship between predicted depression and each stigma/discrimination at varying levels of the coping strategy (±1 SD away from its mean). Other variables and covariates were set at the mean level (categorical variables were set at the reference level). A power analysis was performed for the mixed-effects model using the simr package in R (Green and MacLeod, 2016). Using 1000 simulations at an alpha level of 0.05 and based on 64 participants measured across four time points (*n* = 256 observations), the power analysis revealed an estimated power of 84.90% (95% CI = [82.53, 87.06]) to detect the interaction effect between stigma and coping on depression, with an effect size of 0.22. The results indicated that the study is adequately powered, though slightly below the conventional threshold of 90%.

## 3. Results

### 3.1. Gender identity stigma, coping, and depression

**Fig. 1.**
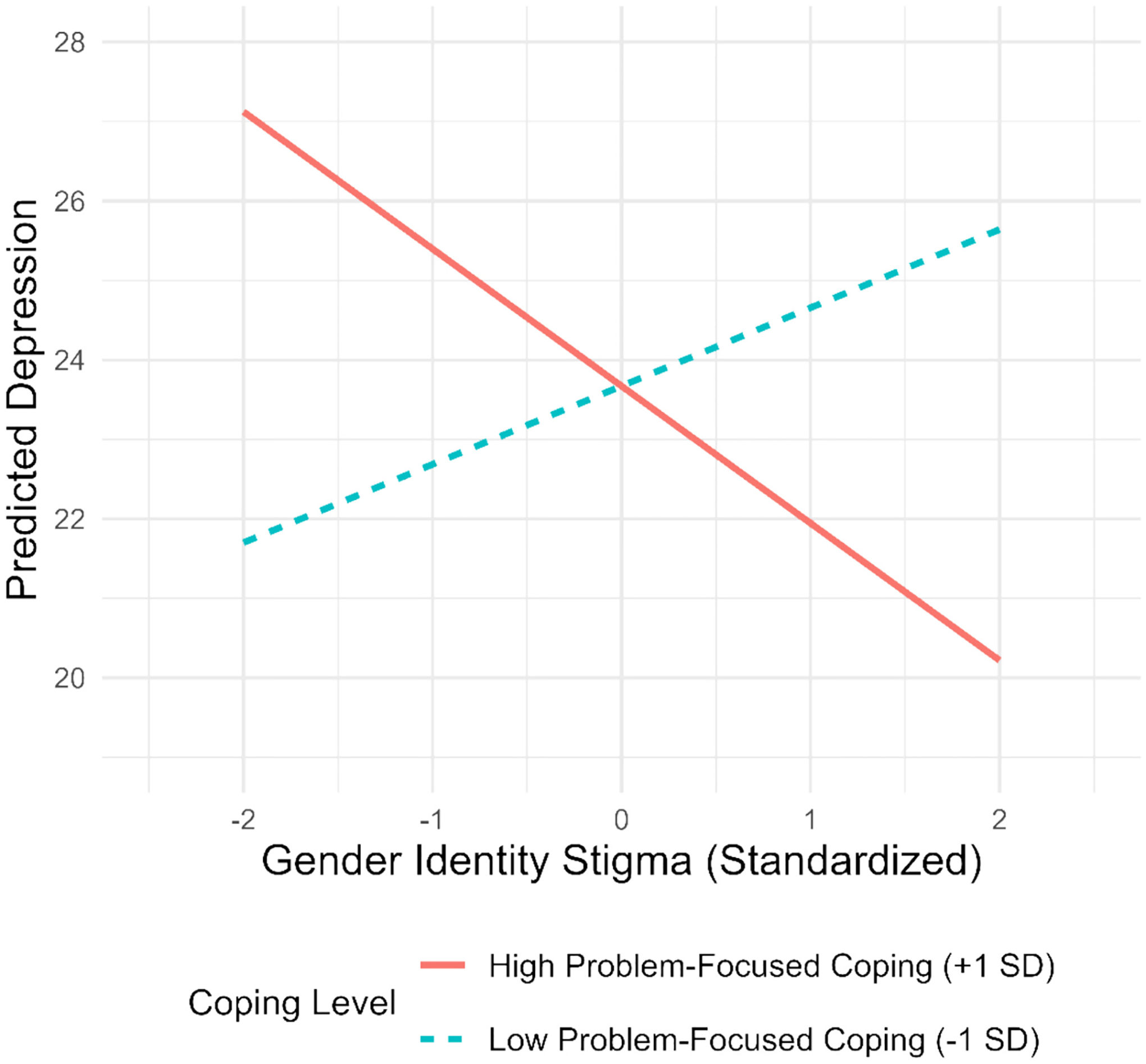
Problem-focused coping mitigates the negative effects of gender identity stigma on depression.

In step 1, gender identity stigma, problem-focused coping, emotion-focused coping, and avoidant coping did not predict depression symptoms after accounting for previous depression, minority status, and group condition (Table 2). We found a significant interaction between gender identity stigma and problem-focused coping in step 2 (*p* = .02). As is shown in Fig. 1, for individuals with low levels of problem-focused coping, a positive relationship between gender identity stigma and subsequent depression (i.e., two months later) was observed, though not at a significant level (*β* = 0.99, *p* > .05). In contrast, for those with high levels of problem-focused coping, experiencing gender identity stigma was associated with lower levels of depression (*β* = − 1.73, *p* < .05).

### 3.2. Discrimination, coping, and depression

**Fig. 2.**
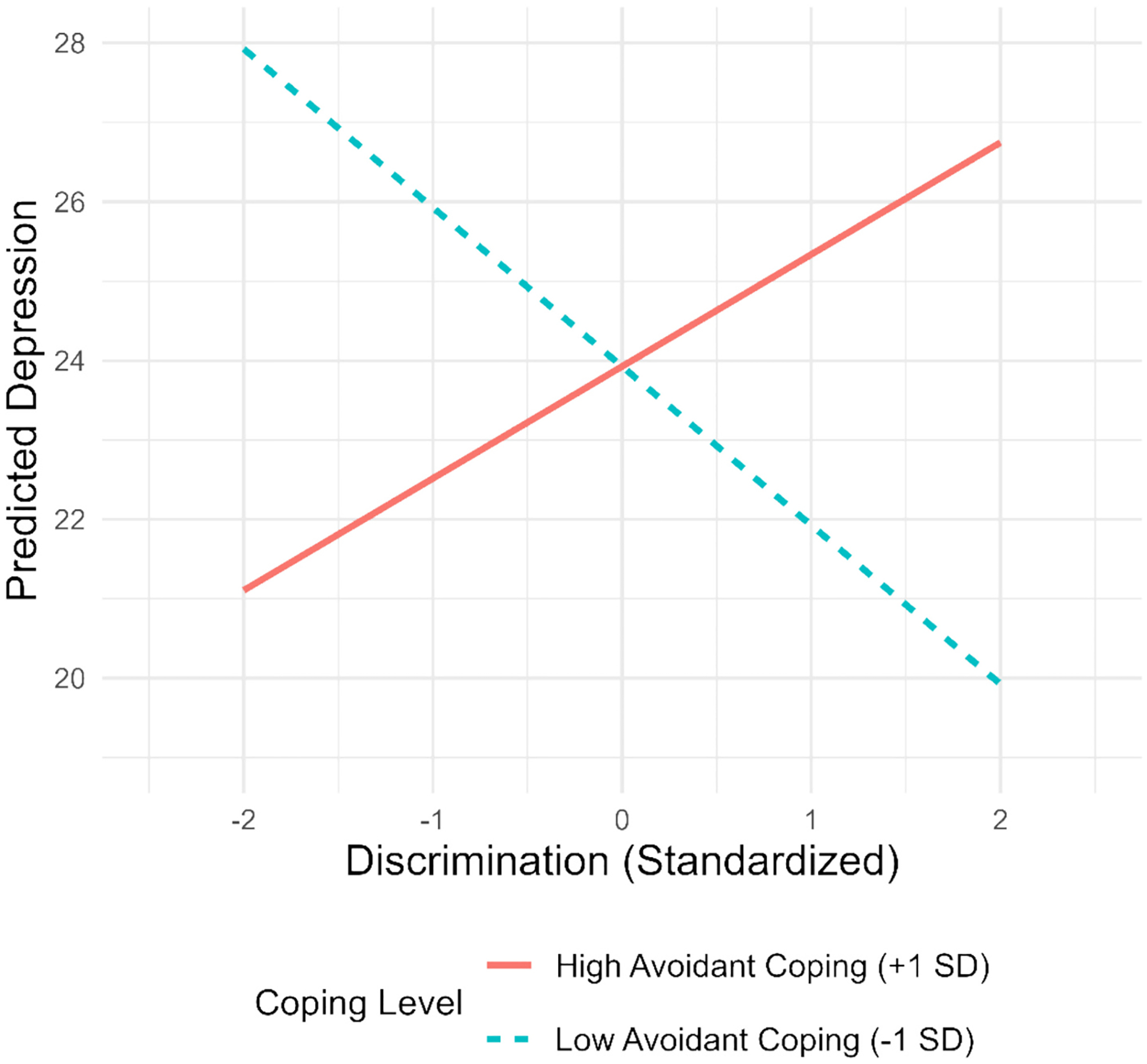
Avoidant coping exacerbates the negative effects of discrimination on depression.

In step 1, discrimination, problem-focused coping, emotion-focused coping, and avoidant coping did not predict depression after accounting for previous depression, minority status, and group condition (Table 3). In the full model, we found a statistically significant interaction between discrimination and avoidant coping (*p* = .010). Specifically, for those with low levels of avoidant coping, experiencing discrimination was associated with lower severity of depression (*β* = − 2.00, *p* < .05). Among individuals with high levels of avoidant coping (+1 SD from the mean), there was a positive, marginally significant trend for an association between discrimination and depression symptoms (*β* = 1.44, *p* = .09). To further probe this pattern, we conducted sensitivity analyses by examining simple slopes at +1.5 SD from the mean of avoidant coping, which revealed a significant association between discrimination and depression (β = 2.27, *p* = .043; see Fig. 2).

### 3.3. Enacted stigma, coping, and depression

After controlling for previous depression, group condition, and minority status, enacted stigma, problem-focused coping, emotion-focused coping, and avoidant coping were not associated with subsequent depression (Table 4). No significant interaction was found between enacted stigma and the three types of coping strategies.

## 4. Discussion

Among EA-SGM, different forms of coping mitigated the impact of stigma on depression. The moderating effects of the three coping strategies varied by type of stigma. Specifically, problem-focused coping mitigated the negative effect of gender identity stigma on depression. Conversely, avoidant coping exacerbated the negative effect of discrimination on future depression. No coping strategies moderated the association between enacted stigma and future depression. These findings suggest that different types of stigma experienced by EA-SGM (Ayhan et al., 2020; Swann et al., 2020) affect the risk for depression differently.

Problem-focused coping, involving direct, action-oriented efforts to manage the stressor, such as planning and seeking support, generally protects against stigma among various minority groups (Chao, 2011; Forster et al., 2022). Our findings provide further evidence that problem-focused coping can buffer the negative effects of gender identity stigma on depression in EA-SGM. For EA-SGM that used little or no problem-focused coping, experiencing gender identity stigma led to greater depression. Conversely, gender identity stigma did not increase risk for depression among those who used more problem-focused coping. Problem-focused coping strategies may help EA-SGM adjust their internalized negative belief, re-evaluate the importance of gender identity on their self-image, and reduce subjective distress (Han et al., 2022). Therefore, if our findings are confirmed, future mental health interventions for EA-SGM might focus on increasing problem-focused coping for those who place high importance on gender identity as their self-concept.

Problem-focused coping did not protect against the effects of discrimination on depression. The goodness-of-fit hypothesis posits that the effects of coping strategies depend on the degree to which the coping strategies are compatible with the controllability of the stressor (Conway and Terry, 1992; Park et al., 2012; Roubinov et al., 2015). Problem-focused coping is more effective in addressing controllable stressors, for which direct actions can mitigate its consequences or address the source (Park et al., 2001, 2012). Unlike gender identity stigma that reflects individuals’ internal experiences of feeling stigmatized due to a marginalized gender status, discrimination stems from external stressors, which are often systemic and interpersonal in nature, making them largely beyond the individual’s control. EA-SGM may encounter this external stigma in various settings (e.g., schools, work-places, & healthcare systems), but lack the resources and support to directly address the source of discrimination (Mitchell et al., 2022). When the discrimination is beyond their ability to change, problem-focused coping may no longer produce favorable outcomes for EA-SGM.

Even if problem-focused coping is ineffective, minimizing the use of avoidant coping can reduce the negative effects of discrimination on EA-SGM. Avoidant coping involving efforts to distance individuals from the stressor such as denying and using substances is generally maladaptive (Chao, 2011; Forster et al., 2022). Consistently, our findings revealed a significant interaction between discrimination and avoidant coping, suggesting that the degree to which EA-SGM uses avoidant coping can alter the negative impact of discrimination. Specifically, for individuals with low levels of avoidant coping, discrimination was associated with lower levels of depression, while for those with high levels of avoidant coping, discrimination was associated with higher levels of depression. Of note, due to the limited sample size, the positive association between discrimination and depression was at a trend level (*p* = .09) for individuals with +1 SD above the mean on avoidant coping, but reached statistical significance for those at +1.5 SD. Future research should probe this relationship using a larger sample. Overall, despite providing temporary relief, avoidant coping often leads to increased distress in the long-term as the stressor remains unresolved and may even worsen (Holahan et al., 2005).

The three coping strategies did not moderate the effects of enacted stigma on depression. Enacted stigma is often considered more severe than other forms of stigma because it involves direct and explicit mistreatment and can result in immediate physical and psychological harm (Swann et al., 2022), especially when it involves violence and victimization. Given the severity and uncontrollability of enacted stigma, it is not surprising that individual-level coping strategies fail to prevent or alter its occurrence and consequences.

Overall, our findings highlight the role of stigma type in shaping effective coping strategies. Despite variations in how stigma is measured and defined across studies, our results suggest that gender identity stigma, as an internalized form of stigma, benefits from problem-focused coping. Conversely, discrimination, an externalized stigma, is better managed with less avoidant coping. Enacted stigma, representing the most severe form of external stigma, appears resistant to the effects of individual-level coping. These distinctions underscore the need for future research to further investigate specific stigma characteristics—particularly its external versus internal focus and severity—and how they influence the appropriateness of coping strategies. A deeper understanding of these relationships could guide the development of tailored interventions that align coping mechanisms with specific stigma characteristics, ultimately enhancing mental health outcomes for marginalized populations.

Our findings support and further refine the Minority Stress Model (Frost and Meyer, 2023; Meyer, 2003). First, while the model categorizes stressors broadly as proximal or distal, our results suggest a more comprehensive classification based on the controllability of the stressor. Second, the findings underscore the importance of stressor–coping fit. Consistent with the goodness-of-fit hypothesis, our results suggest that the effectiveness of a coping strategy depends on the controllability and locus of the stressors. Third, the results underscore the importance of coping flexibility in addressing multiple stigmas experienced by minorities. Future researchers may consider incorporating coping flexibility as a moderator of the stress-health relationship in the Minority Stress Model.

### 4.1. Clinical implications

Our findings can inform more targeted interventions for EA-SGM populations. A key implication is the importance of coping flexibility, the ability to adapt coping strategies to the nature of the stressor (Cheng et al., 2014). Beyond offering a wide set of coping strategies, future mental health interventions for minority populations could benefit from training individuals to select appropriate coping strategies based on the type of stigma they face. For instance, programs could include skills training to appraise whether a stressor is controllable, for example, distinguishing between internalized, controllable stigma (e.g., gender identity stigma) and external, uncontrollable stigma (e.g., being physically assaulted by strangers), and teach suitable coping strategies.

For controllable stressors, interventions should focus on problem-focused coping strategies, including identity-affirming cognitive restructuring, advocacy, and self-acceptance. For uncontrollable stressors, interventions can emphasize the maladaptive nature of avoidant coping and assist individuals in identifying and adopting alternative methods of coping. Interventions should also train individuals to seek group- and institutional-level support, such as accessing LGBTQ communities, when individual-level coping is not effective. Furthermore, clinicians can help individuals reflect on the effectiveness of coping strategies via self-monitoring tools and refine their coping approaches over time. Personalized approaches that consider the variability in stressor types and coping responses can better support the mental health of this vulnerable population.

### 4.2. Limitations and future directions

There are several limitations to consider when interpreting the current findings. First, given the relatively small sample size and the somewhat limited statistical power (84.9 %), the findings should be interpreted as exploratory. Likewise, the sample was recruited based on suicidal ideation in a clinical trial of a suicide prevention intervention and therefore may not fully represent the broader EA-SGM population. Rather, this sample may be more similar to a clinical sample and therefore experience higher levels of distress and adopt more maladaptive coping mechanism compared to the non-clinical population. To increase the generalizability of the current findings, it is important to replicate the current study using a larger, more diverse sample. Second, while this study aimed to examine the moderating effects of three coping strategies for different types of stigma, the coping strategies were assessed as a general tendency using the Brief-COPE rather than in relation to specific types of stigma. This approach assumes that EA-SGM applies the same coping strategies consistently across different forms of stigma; however, their coping strategies may vary depending on the nature of the stressor. For example, one may adopt more avoidant coping when facing enacted stigma but use problem-focused coping to address gender identity stigma. Furthermore, some may demonstrate greater flexibility in tailoring their coping strategies to specific stigma experiences than others. To better assess the distinct effects of different coping strategies and better inform the match of coping strategies to address specific types of stigma, future research should consider assessing individuals’ use of different coping strategies for each form of stigma and examine the role of coping flexibility.

Third, although one advantage of the current study is the longitudinal design and time-lagged analysis, our findings were limited to the short-term effects of stigma (i.e., 2 months). The longer-term impacts of stigma on depression and the moderating role of coping strategies remain unknown. Experiences of stigma tend to be chronic for EA-SGM (Ayhan et al., 2020; Valdiserri et al., 2019). The cumulative exposure to stigma over time is likely to have a more profound impact on depression, while this impact may be less visible in the short term, especially when previous depression is controlled for in the current analysis. This may explain why the main effects of discrimination, gender identity stigma, and enacted stigma on future depression were not statistically significant. Furthermore, chronic exposure to stigma might require different coping strategies than acute or short-term stigma. Indeed, the fact that stigma is chronic suggests that it is unlikely to be resolved. Thus, problem-focused coping, which is typically effective for controllable stressors (Park et al., 2001, 2012), might become less protective over time in this context. Therefore, it may be worthwhile to examine the impacts of chronic or cumulative exposure to stigma and explore the moderating effects of coping in the longer term.

Furthermore, the current study only examined three coping strategies. Other protective factors and risk factors, such as social support and community resources, could be explored. Finally, the measure of gender identity stigma used in this study was adapted to better reflect the experiences of EA-SGM. While the adapted measure demonstrated acceptable internal consistency, we did not include other measures of gender identity stigma to formally assess its convergent validity. Future research should validate this stigma measure by examining its associations with relevant instruments.

## 5. Conclusion

This study was the first to examine the moderating roles of three coping strategies on the associations between multiple types of stigma and depression among EA-SGM. The findings highlight that the effectiveness of coping strategies depends on the nature of the stigma. In this study, problem-focused coping is effective in dealing with gender identity stigma but does not yield protective effects for discrimination or enacted stigma. Avoidant coping is particularly harmful in the context of discrimination. Given that minority groups such as EA-SGM often face multiple types of stigma (Ayhan et al., 2020; Swann et al., 2020), which may not be addressed by one-size-fits-all strategies, future interventions should help EA-SGMs flexibly adjust their coping strategies to the specific stressors and explore additional resources when individual coping strategies are not effective.

## Supplementary Material

1

Supplementary data to this article can be found online at https://doi.org/10.1016/j.jad.2025.119843.

## Figures and Tables

**Table 1:** Sample characteristics.

Variables	*M*	*SD*
Age	36.66	13.38
	*n*	*%*
Gender identity		
Cisgender man	8	12.50
Cisgender women	24	37.50
Transgender woman	3	4.69
Transgender man	3	4.69
Genderqueer	8	12.50
Non-binary	13	20.31
Questioning	2	3.13
Agender	1	1.56
Other	2	3.13
Race		
White	32	50.00
Asian	19	29.69
Black	7	10.94
Biracial or multiracial	4	6.25
Other	2	3.13
Ethnicity		
Non-Hispanic	55	85.94
Hispanic	9	14.06
Sexual identity		
Gay	12	18.75
Lesbian	11	17.19
Bisexual	28	43.75
Queer	7	10.94
Asexual	2	3.13
Other	4	6.25

**Table 2:** Mixed effect model of gender identity stigma, coping strategies and their interactions on depression.

	Unconditional model without interaction terms			Full model with interaction terms		
	*β*	*SE*	*p*	*β*	*SE*	*p*
Intercept	15.37	2.58	<0.001***	17.27	3.19	<0.001***
Depression	0.35	0.08	<0.001***	0.23	0.08	0.01**
Group condition (0 = Control; 1 = Intervention)	0.55	1.01	0.60	0.76	1.12	0.50
Minority score	0.15	0.58	0.80	0.26	0.64	0.69
Gender identity stigma	−0.50	0.48	0.31	−0.33	0.53	0.53
Problem-focused coping	−0.89	0.60	0.17	−0.97	0.66	0.15
Emotion focused coping	0.49	0.59	0.42	0.62	0.65	0.35
Avoidant coping	0.71	0.52	0.20	0.67	0.57	0.26
Gender identity stigma X problem-focused coping				−1.36	0.55	0.02*
Gender identity stigma X emotion focused coping				0.46	0.57	0.42
Gender identity stigma X avoidant coping				−0.67	0.44	0.13

Note.

* *p* < .05.

** *p* < .01.

*** *p* < .001.

**Table 3:** Mixed effect model of discrimination, coping strategies and their interactions on depression.

	Unconditional model without interaction terms			Full model with interaction terms		
	*β*	*SE*	*p*	*β*	*SE*	*p*
Intercept	13.23	2.42	<0.001***	16.71	3.13	<0.001***
Depression	0.45	0.08	<0.001***	0.31	0.08	<0.001***
Group condition (0 = Control; 1 = Intervention)	0.61	0.92	0.52	0.66	1.07	0.55
Minority score	−0.07	0.52	0.90	−0.28	0.62	0.66
Discrimination	0.03	0.48	0.95	−0.33	0.51	0.51
Problem-focused coping	−1.02	0.53	0.09	−1.12	0.64	0.11
Emotion-focused coping	0.54	0.52	0.33	0.57	0.61	0.37
Avoidant coping	0.42	0.47	0.40	0.54	0.55	0.35
Discrimination X Problem-focused coping				0.95	0.86	0.27
Discrimination X Emotion-focused coping				−0.65	0.78	0.41
Discrimination X Avoidant coping				1.71	0.65	0.01*

Note.

* *p* < .05.

*** *p* < .001.

**Table 4:** Mixed effect model of enacted stigma, coping strategies and their interactions on depression.

	Unconditional model without interaction terms			Full model with interaction terms		
	*β*	*SE*	*p*	*β*	*SE*	*p*
Intercept	13.84	2.51	<0.001***	14.27	2.99	<0.001***
Depression	0.43	0.08	<0.001***	0.40	0.08	<0.001***
Group condition (0 = Control; 1 = Intervention)	0.63	0.96	0.53	0.46	1.00	0.66
Minority Score	−0.03	0.54	0.96	−0.08	0.56	0.89
Enacted Stigma	−0.27	0.43	0.53	−0.28	0.44	0.53
Problem-focused coping	−1.02	0.55	0.10	−1.04	0.58	0.10
Emotion-focused coping	0.50	0.55	0.38	0.52	0.57	0.38
Avoidant coping	0.43	0.49	0.40	0.58	0.52	0.29
Enacted Stigma: Problem-focused coping				−0.49	0.40	0.22
Enacted Stigma: Emotion-focused coping				0.06	0.52	0.90
Enacted Stigma: Avoidant coping				0.49	0.50	0.32

Note.

*** *p* < .001.
