# Supplementary material for: Moderating effects of coping strategies on the associations between stigma and depression among emerging adults
Source: J Affect Disord. Author manuscript; Available in PMC 2026 Apr 9. (PMC13064578; doi:10.1016/j.jad.2025.119843)
Supplement: 1 [file NIHMS2162353-supplement-1.docx]

**Supplemental Table 1**

Correlation Matrix

|  | 1 | 2 | 3 | 4 | 5 | 6 | 7 | 8 | 9 | *Mean* | *SD* |
| --- | --- | --- | --- | --- | --- | --- | --- | --- | --- | --- | --- |
| 1. Group condition  (0 = Control; 1= Intervention) | 1.00 | -0.12 | -0.06 | 0.02 | 0.02 | -0.05 | 0.04 | 0.09 | -0.13 | 0.50 | 0.50 |
| 2. Minority Score | -0.12 | 1.00 | -0.13 | 0.12 | 0.08 | 0.18 | 0.18 | -0.02 | 0.03 | 2.08 | 0.88 |
| 3. Gender identity stigma | -0.06 | -0.13 | 1.00 | -0.03 | -0.27 | -0.15 | 0.03 | 0.16 | -0.22 | 2.98 | 0.60 |
| 4. Discrimination | 0.02 | 0.12 | -0.03 | 1.00 | 0.37 | 0.10 | -0.03 | 0.04 | 0.02 | 1.20 | 0.38 |
| 5. Enacted Stigma | 0.02 | 0.08 | -0.27 | 0.37 | 1.00 | 0.10 | 0.06 | 0.13 | 0.19 | 1.72 | 0.56 |
| 6. Problem-focused coping | -0.05 | 0.18 | -0.15 | 0.10 | 0.10 | 1.00 | 0.49 | -0.12 | -0.12 | 2.67 | 0.66 |
| 7. Emotion focused coping | 0.04 | 0.18 | 0.03 | -0.03 | 0.06 | 0.49 | 1.00 | 0.13 | 0.09 | 2.84 | 0.44 |
| 8. Avoidant coping | 0.09 | -0.02 | 0.16 | 0.04 | 0.13 | -0.12 | 0.13 | 1.00 | 0.18 | 2.47 | 0.61 |
| 9. Depression | -0.13 | 0.03 | -0.22 | 0.02 | 0.19 | -0.12 | 0.09 | 0.18 | 1.00 | 26.11 | 6.07 |
